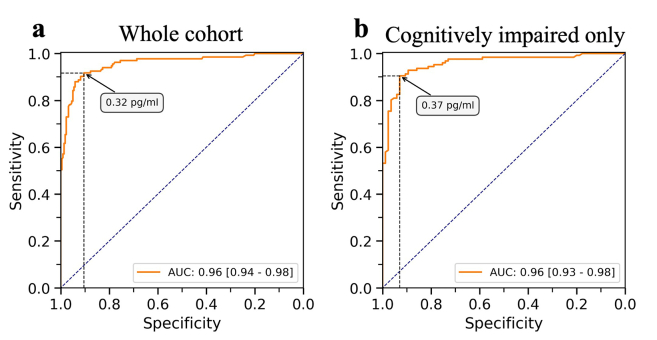# Detection and staging of Alzheimer's disease by plasma pTau217 on a high throughput immunoassay platform

**DOI:** 10.1016/j.ebiom.2024.105405

**Published:** 2024-10-21

**Authors:** Azadeh Feizpour, James David Doecke, Vincent Doré, Natasha Krishnadas, Kun Huang, Pierrick Bourgeat, Simon Matthew Laws, Christopher Fowler, Joanne Robertson, Lucy Mackintosh, Scott Ayton, Ralph Martins, Stephanie Ruth Rainey-Smith, Kevin Taddei, Larry Ward, Eddie Stage, Anthony Wilson Bannon, Colin Louis Masters, Jurgen Fripp, Victor Luis Villemagne, Christopher Cleon Rowe

**Affiliations:** a The Florey Institute of Neuroscience and Mental Health, The University of Melbourne, Parkville, Victoria, Australia; b Department of Molecular Imaging & Therapy, Austin Health, Melbourne, Victoria, Australia; c The Australian e-Health Research Centre, CSIRO, Brisbane, Queensland, Australia; d Centre for Precision Health, Edith Cowan University, Joondalup, Western Australia, Australia; e The Australian e-Health Research Centre, CSIRO, Melbourne, Victoria, Australia; f Collaborative Genomics and Translation Group, Edith Cowan University, Joondalup, Western Australia, Australia; g Curtin Medical School, Curtin University, Bentley, Western Australia, Australia; h Australian Alzheimer's Research Foundation, Nedlands, Perth, Australia; i Centre for Healthy Ageing, Health Futures Institute, Murdoch University, Murdoch, Western Australia, Australia; j AbbVie, North Chicago, IL, USA; k Department of Psychiatry, University of Pittsburgh, Pittsburgh, PA, USA; l Florey Department of Neuroscience and Mental Health, The University of Melbourne, Melbourne, Victoria, Australia

**Keywords:** Alzheimer's disease, Plasma biomarker, Phospho-tau, pTau217, Positron emission tomography, PET

## Abstract

**Background:**

Plasma phospho-tau 217 (pTau217) assays can accurately detect Alzheimer's disease (AD) pathology, but clinical application is limited by the need for specialised equipment. This study tests the performance of a plasma pTau217 assay performed on the Lumipulse-G® platform, that is in widespread clinical use, for selecting patients for therapy based on β-amyloid (Aβ) status and tau staging.

**Methods:**

Participants included 388 individuals with ^18^F-NAV4694 Aβ-PET and ^18^F-MK6240 tau-PET. Association of pTau217 with PET was examined using Spearman's correlation. Discriminative performance for Aβ and tau PET status as well as tau staging was assessed using Receiver Operating Characteristic analysis.

**Findings:**

Plasma pTau217 had a high correlation with both Aβ Centiloid (*r* = 0.76) and tau SUVR_meta-temporal_ (*r* = 0.78). Area under curve (AUC) was 0.93 for Aβ− vs Aβ+ and 0.94 for tau− vs tau+. Applying one threshold (Youden's index), pTau217 was 87% accurate in classification of participants to Aβ− vs Aβ+. Applying two thresholds to classify participants into Low, Indeterminate, and High zones, 17.8% had Indeterminate results and among Low/High zone participants, 92% were correctly classified as Aβ− or Aβ+. The assay accurately discriminated moderate/high neocortical tau from no tau or tau limited to mesial-temporal lobe (AUC 0.97) and high neocortical tau from all others (AUC 0.94).

**Interpretation:**

Plasma pTau217, measured by the widely-available, fully-automated Lumipulse®, was a strong predictor of both Aβ and tau PET status and demonstrated strong predictive power in identifying individuals likely to benefit the most from anti-Aβ treatments.

**Funding:**

10.13039/501100000925NHMRC grants 1132604, 1140853, 1152623 and 10.13039/100006483AbbVie.

**Research in context**

**Evidence before this study**

We searched research articles on PubMed written between years 2018 and 2024, with the keywords “Alzheimer's disease”, “plasma pTau217”, “plasma phospho-tau 217”. There were no language restrictions. The present literature suggests that among plasma biomarkers for Alzheimer's disease (AD), plasma pTau217 exhibits the most promising performance in detecting AD pathology as assessed through CSF or PET measurements. However, performance varies depending on the specific method and antibodies employed, resulting in inter-lab variabilities, and most studies used platforms approved for research use only restricting potential for widespread clinical use. Recently, it has been proposed that AD be staged into initial, early, intermediate, or advanced stages based on the presence of β-amyloid (Aβ) and the topographic extent and magnitude of tau aggregation using both Aβ and tau PET. Advanced biological disease stage adversely influences the benefit achieved from Aβ monoclonal antibody therapy.

**Added value of this study**

In this study, we show that plasma pTau217 correlates strongly with PET measures of aggregated tau across the clinical spectrum of AD and can discriminate individuals at intermediate or advanced stage vs lower stages of AD. This was accomplished while maintaining high accuracy in correct classification of participants to PET Aβ− vs Aβ+. Furthermore, the study demonstrated that measurement of plasma pTau217 can be performed accurately on a commercial, fully automated, high throughput immunoassay platform, the Lumipulse G, an FDA approved device for CSF AD biomarker measurement.

**Implications of all the available evidence**

Firstly, highly accurate measures of plasma pTau217 not only predict brain Aβ and tau but the plasma level also reflects the topographic extent and magnitude of tau aggregation. Plasma pTau217 is not only useful to assist early and accurate diagnosis of AD but may also contribute to prognosis and more tailored selection for Aβ monoclonal antibody therapies where benefit appears reduced in persons with advanced tau aggregation. Secondly, the Lumipulse G platform has a large global installation base in clinical pathology services, that will facilitate access and uptake of plasma pTau217 measures into clinical practice.

## Introduction

In recent years, Alzheimer's disease (AD) research has experienced a pivotal breakthrough with the emergence of increasingly sensitive plasma biomarker assays. The significance of these biomarkers becomes evident in settings where access to advanced diagnostic tools such as positron emission tomography (PET) is limited or cost-prohibitive. This becomes particularly crucial in the current era of disease-modifying therapies, where the availability of these blood tests could facilitate earlier diagnosis, improving the screening process for clinical trials and enhancing access to, and outcomes from disease-modifying therapies. Additionally, there is evidence suggesting that individuals with low-medium tau levels, determined via PET, respond more favourably to β-amyloid (Aβ) antibody therapy than those with high tau,^1^ highlighting a need to establish whether plasma pTau assays can provide this information.

Among plasma biomarkers, tau phosphorylated at different epitopes has been shown to hold promise as biomarkers of AD pathologies, with plasma tau phosphorylated at threonine 217 (pTau217) showing the best performance to date.^2^, ^3^, ^4^, ^5^, ^6^ This performance varies by assay and capture and detection antibodies used, with best correlation reported to date to Aβ PET of *r* = 0.74,^7^ and tau PET of *r* = 0.78.^8^

A Lumipulse based assay was recently developed for measuring plasma levels of pTau217.^9^ The Lumipulse assay is fully automated and compared to many of the assay platforms used in previous pTau studies, offers more consistent performance, high throughput, easy access and availability and an established installation base in clinical services world-wide.

In this cross-sectional study, we investigated the diagnostic performance of the Lumipulse G pTau217 plasma assay in participants from the Australian Imaging, Biomarkers and Lifestyle study of ageing (AIBL) and the Australian Dementia Network (ADNeT) cohorts. We evaluated the performance of this pTau217 assay against the latest generation Aβ and tau PET agents and assessed the association of pTau217 with cerebral Aβ and tau burden, as measured by PET. In addition, we assessed the clinical utility of pTau217 by examining its distribution across PET-based Braak stages, as well as the 2024 revised criteria for staging AD based on PET imaging.^10^

## Methods

### Participants

Participants from AIBL and ADNeT cohorts (n = 388) with matched plasma pTau217, ^18^F-NAV4694 Aβ PET, and ^18^F-MK6240 tau PET were included. Information pertaining to AIBL cohort recruitment and evaluation are outlined in a previous report.^11^ The ADNeT cohort participants were evaluated as per AIBL criteria. Classification of participants to cognitively unimpaired (CU), mild cognitive impairment (MCI) and dementia was performed by a multi-disciplinary panel blind to PET imaging and blood assays results. This panel comprised old age psychiatrists, neuropsychologists, psychologists, and at times a neurologist and geriatrician. Participants with neuropsychological assessment performance within 1.5 standard deviation (SD) of the published norms for their age group, were classified as CU. A classification of MCI or dementia was determined based on internationally agreed clinical criteria.^12^^,^^13^ Participants included 156 CU, 100 with MCI and 132 with dementia. For identifying sex, study participants were required to self-report.

### Ethics

AIBL study was approved by the institutional ethical review committee at St. Vincent's Hospital, Melbourne (SAGE Project ID Number: 2022/PID06188; SVHM Local Ref ID: HREC 028/06) and ADNeT study was approved by the institutional ethical review committee at Austin Health, Melbourne (Project Number: HREC/59189/Austin-2019; Austin Health SSA Reference Number: SSA/59189/Austin-2020). Written informed consents were obtained from all participants, prior to undergoing study procedures.

### PET image acquisition

The Aβ PET imaging involved a 20-min acquisition, carried out 50 min after intravenous injection of 200 MBq of ^18^F-NAV4694. The tau PET imaging was performed on a different day, involving a 20-min acquisition, and conducted 90 min after intravenous administration of 185 MBq of ^18^F-MK6240. Spatial normalization for Aβ PET scans employed the CapAIBL method,^14^ followed by standardisation using the Centiloid (CL) scale,^15^^,^^16^ which used the standard whole cerebellum mask as the reference region. Throughout this study we utilised an Aβ PET positivity (Aβ+) threshold of ≥25 CL. However, we also investigated the use of additional Aβ thresholds between 10 and 50 CL.

**Supplementary Figure S1:**
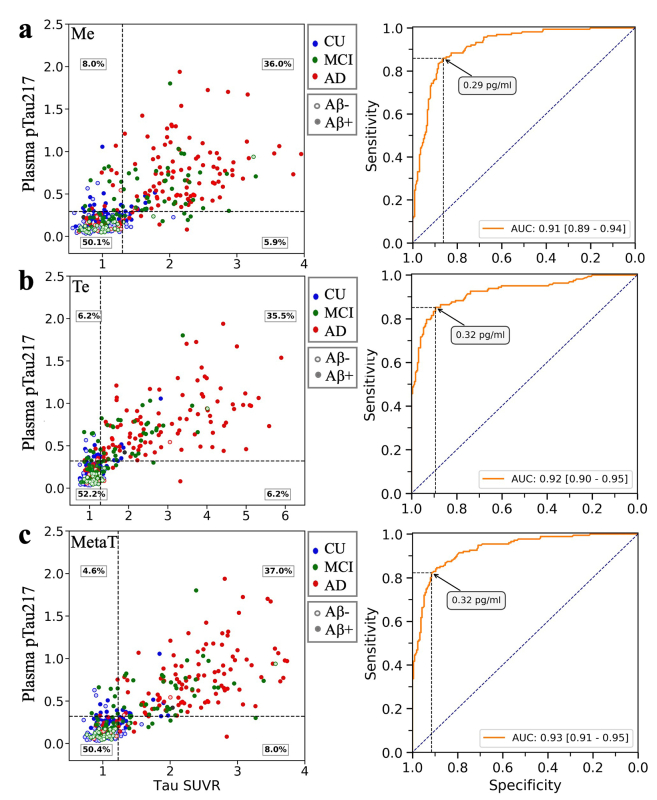


For tau PET scans, spatial normalization was achieved using the MR-less CapAIBL PCA-based method,^17^ and tau PET scans were scaled using the mid-cerebellar cortex as the reference region. ^18^F-MK6240 tau standardised uptake value ratio (SUVR) were computed in composite regions of interest (ROIs): mesial temporal (Me) ROI comprised entorhinal cortex, hippocampus, parahippocampus and amygdala; temporoparietal (Te) ROI consisted of inferior temporal, fusiform, supramarginal and angular gyri, posterior cingulate/precuneus, superior and inferior parietal, and lateral occipital cortex; the rest of neocortex (R) ROI included dorsolateral and ventrolateral prefrontal, orbitofrontal, gyrus rectus, superior temporal, and anterior cingulate cortex,^18^ and finally meta temporal (MetaT) ROI, consisted of the Me ROI as well as inferior and middle temporal and fusiform gyri. Tau positivity (tau+), in each ROI, was primarily defined based on the 2.5 standard deviation (SD) from the mean SUVR in the Aβ− CU. However, 95th percentile thresholds were also examined for some analyses (specifically, determining ^18^F-MK6240 tau PET status in Supplementary Figure S1). The ^18^F-MK6240 tau SUVR were also computed in CapAIBL ROIs derived from Freesurfer Desikan-Killiany Atlas,^19^ corresponding to the different Braak stages.

### PET-based Braak stage definition

The stages were defined as follows: Braak I (entorhinal), Braak II (Hippocampus), Braak III (parahippocampal, fusiform, lingual, Amygdala), Braak IV (middle temporal, thalamus-proper, caudal anterior cingulate, rostral anterior cingulate, posterior cingulate, isthmus cingulate, insula, inferior temporal and temporal pole), Braak V (superior frontal, lateral orbitofrontal, medial orbitofrontal, frontal pole, caudal middle frontal, rostral middle frontal, parsopercularis, parsorbitalis, parstriangularis, caudate, putamen, lateral occipital, supramarginal, inferior parietal, superior parietal, superior temporal, pallidum, precuneus, bankssts, accumbens, transverse temporal), and Braak VI (pericalcarine, postcentral, cuneus, precentral, paracentral).

### PET-based Braak stage assignment

Positivity in each Braak region was defined based on the 2.5 standard deviation (SD) of the mean SUVR in the Aβ− CU group, after excluding cases with primary age-related tauopathy (PART). PET-based Braak stage was then assigned by the “latest” positive Braak region given that earlier regions were also positive. Braak 0 participants were tau negative within each Braak region. Participants with positive Braak regions out of the expected order were categorised as ‘Atypical’ and displayed in the boxplots but not included in Braak stage Receiver Operating Characteristic (ROC) analyses. As per the B score in the Alzheimer's disease neuropathological criteria (ADNC), Braak stages were grouped as I–II, III–IV, and V–VI for statistical comparisons. For the ROC analyses, PET-based Braak stages were dichotomised to low and high, with low tau defined as Braak 0–III and high Braak tau defined as Braak IV–VI.

### Biological PET stage assignment

The assignment of participants to biological PET stages followed the recommendation by the Revised Criteria for Diagnosis and Staging of Alzheimer's Disease (2024).^10^ Briefly, participants were assigned to ‘Initial’ (A+T−), ‘Early’ (A+T_MTL_+), ‘Intermediate’ (A+T_MOD_+), or ‘Advanced’ stage (A+T_HIGH_+) where A+ denoted Aβ+ based on a Centiloid threshold of 25 CL, T- denoted tau negative in all ROIs, T_MTL_+ indicated tau uptake limited to the medial temporal lobe (Me ROI), T_MOD_+ represented *moderate* tau uptake in Te ROI and T_HIGH_+ indicated *high* tau uptake in Te ROI. A Te threshold of 2.68 SUVR between T_MOD_ and T_HIGH_ was derived using the method from the Trailblazer-Alz 2 randomised clinical trial.^1^ This threshold was achieved by defining High tau burden as the upper quartile of tau PET results in the Aβ+ MCI/AD participants within the entire AIBL study who have undergone ^18^F-MK6240 tau PET imaging (n = 348). The participants failing to satisfy these criteria were categorised as ‘Atypical’ and displayed in the boxplots but not included in ROC analyses. Considering that tau positivity thresholds based on 95th percentile of Aβ− CU group yielded 22 Atypical cases while the thresholds based on mean + 2.5 SD of Aβ− CU group gave only 5 Atypical cases, biological PET staging was only performed based on the latter thresholds.

### Plasma pTau217 assays

Fasted blood samples were collected from participants 0.7 ± 5.7 months from the time of the Aβ PET scan and 0.3 ± 3.2 months from the time of tau PET scan. Plasma samples were stored in K2-EDTA tubes (7.5 ml S-monovette 01.1605.008, Sarstedt) with pre-added prostaglandin E1 (33 ng/ml of whole blood, Sapphire Biosciences) which prevented platelet activation. Samples were centrifuged at room temperature at 200 g for 10 min to collect platelet-rich plasma. Subsequently, they were centrifuged at 800 g for 10 min to obtain plasma. The extracted plasma was snap frozen within 2 h of collection and stored in vapor phase liquid nitrogen. Sample aliquots were then shipped on dry ice from Australia to Fujirebio Inc facilities (Malvern, Pennsylvania, USA). Samples were tested on a LUMIPULSE G1200 instrument (with a single freeze thaw cycle), using the Lumipulse G pTau217 Plasma (RUO) assay (lot number D4C4025). One hundred μL of plasma was used for concentration determination, with each sample undergoing a single replicate test.

### Statistics

Data analyses were performed using Python (version 3.9.13) and statistical analyses were performed using statsmodels library (version 0.13.5), scipy (version 1.7.1) and sklearn (version 1.2.1). Our power analysis confirmed that a minimum sample size of 15 per subgroup was adequate to attain 90% power for detecting a between-group difference with effect size of ≥1.5. Normality of residuals was assessed using the Shapiro–Wilk test. Between-group comparisons for quantitative features were performed using one-way analysis of variance (ANOVA) where residuals were normally distributed or the Kruskal–Wallis test where non-normally distributed, followed by Tukey's Honest Significant Difference (HSD) with family-wise error rate (FWER) <0.05. Categorical (Sex, Apolipoprotein E (*APOE*) ε4 allele carriership, Aβ PET+, and MetaT tau PET+) comparisons were conducted with the chi-square (χ^2^) test. Values are reported as mean ± standard deviation (SD) when normally distributed and median (interquartile range (IQR)) when skewed. The correlation between pTau217 and Aβ CL or tau SUVR was evaluated using Spearman's correlation (r). To investigate the ability of pTau217 to discriminate between groups, ROC analyses were employed. To investigate the ability of pTau217 in combination with other factors, such as age and or sex, to discriminate between groups, logistic regression models were employed to calculate the predicted probability of the outcome measure, prior to conducting ROC analyses. Optimal plasma pTau217 thresholds for discriminating between the groups were defined using Youden's index, or cut-off points set at defined sensitivity and specificity levels. For area under curve (AUC), specificity, sensitivity, positive predictive values (PPV), negative predictive values (NPV), accuracy, and correlation coefficients, the 95% confidence intervals (95% CI, shown in square brackets) were calculated by using 1000 bootstrap replicates (without replacement). The 95% CI were then computed using the percentile method. The NPV and PPV were not adjusted for disease prevalence. PET Aβ+ probability was derived from a logistic regression model including plasma pTau217. The linearity assumption underlying logistic regression model for quantitative predictors was assessed using a scatter plot and was met. A two-threshold approach was assessed using pTau217 thresholds set at sensitivity and specificity of 90% or 95%. This approach classified the participants to Low, Indeterminate and High zones for Aβ PET positivity. Percentage of High zone participants who were PET Aβ+ and Low zone participants who were PET Aβ− were reported. The overall correct classification was computed as the percentage agreement between plasma pTau217 and Aβ PET status in the Low and High zones, *excluding the Indeterminate cases*. When discriminating between PET-based Braak stages or biological PET stages, AUC values from different models were compared using DeLong's test. All *p* values were corrected for multiple comparisons (Bonferroni correction or Tukey HSD with family-wise error rate (FWER) <0.05) and reported as “adjusted *p*”.

### Role of funders

Fujirebio performed the pTau217 assay blind to participant information and sent the results back to AIBL investigators for analysis. The co-authors employed by Abbvie had no role in study design, data analysis, data interpretation, or writing of the report. However, they provided comments on the draft manuscript and approval for its submission. The other sponsors had no role in the design and conduct of the study, in the collection, analysis, and interpretation of data, or in the preparation of the manuscript.

## Results

### Demographic characteristics

A total of 388 participants were included in this study: 156 CU, 100 with MCI and 132 with dementia (Table 1). Disease prevalence defined by Aβ PET status was 64% overall, 40% in CU, 69% in MCI and 89% in those with dementia. Participants with dementia were on average younger and had lower median education levels than CU participants and those with MCI. The ratio of males to females was similar across the three clinical groups. As expected, *APOE* ε4 carriership, clinical dementia rating sum of boxes (CDR SoB), brain Aβ burden, brain tau burden (MetaT) and plasma concentration of pTau217 differed by group and were highest in the dementia group and lowest in the Aβ− CU group. For a breakdown by clinical groups (CU, MCI and dementia) and their statistical comparisons, see Supplementary Table S1. For a breakdown by biological PET stages, see Supplementary Table S2.

**Table 1:** Demographics characteristics for clinical groups stratified by Aβ PET status.

	CU Aβ−	CU Aβ+	MCI Aβ−	MCI Aβ+	Dementia Aβ−	AD Aβ+
Sample size	94	62	31	69	15	117
Age (years), mean ± SD	74.7 ± 4.7	76.2 ± 6.6	69.6 ± 8.2	75.5 ± 6.9	71.4 ± 6.6	70.0 ± 7.9
Education (years), median (IQR)	15.0 (12.0–16.0)	12.0 (11.0–15.0)	12.0 (11.0–15.0)	12.0 (10.0–15.0)	11.0 (9.5–12.0)	11.0 (10.0–15.0)
Sex						
Male, n (%)	48 (51)	29 (47)	15 (48)	41 (59)	10 (67)	59 (50)
Female, n (%)	46 (49)	33 (53)	16 (52)	28 (41)	5 (33)	58 (50)
APOE ε4+ (%)	24	56	29	72	13	74
MMSE, median (IQR)	29.0 (28.0–30.0)	29.0 (27.0–30.0)	27.0 (26.0–29.0)	26.0 (24.0–27.0)	23.0 (21.0–24.5)	23.0 (20.0–24.0)
CDR SoB, median (IQR)	0.0 (0.0–0.0)	0.0 (0.0–0.0)	0.5 (0.5–1.4)	1.5 (1.0–2.0)	4.0 (3.8–5.0)	4.0 (3.5–5.0)
Centiloid, median (IQR)	−0.3 (−4.6 to 7.7)	79.3 (48.1–117.7)	−0.9 (−4.9 to 5.9)	113.0 (83.0–140.2)	2.5 (−3.5 to 9.6)	113.4 (89.4–138.3)
MK6240 SUVR_MetaT_, median (IQR)	1.0 (0.9–1.0)	1.1 (1.0–1.3)	1.0 (0.9–1.1)	1.4 (1.1–2.0)	1.0 (1.0–1.2)	2.2 (1.6–2.8)
Plasma pTau217, pg/ml, median (IQR)	0.1 (0.1–0.1)	0.2 (0.2–0.3)	0.1 (0.1–0.1)	0.4 (0.2–0.7)	0.1 (0.1–0.2)	0.6 (0.4–0.9)

CU, cognitively unimpaired; MCI, mild cognitive impairment; AD, Alzheimer's disease; Aβ, β-Amyloid; SD, standard deviation; *APOE* ε4+, Apolipoprotein E ε4 positive; MMSE, Mini-Mental State Examination; CDR-SoB, Clinical Dementia Rating Scale Sum of Boxes; SUVR_MetaT_, Standardised Uptake Value Ratio in the meta temporal region; IQR, interquartile range.

### Plasma pTau217 concentration in clinical groups by Aβ status

**Fig. 1:**
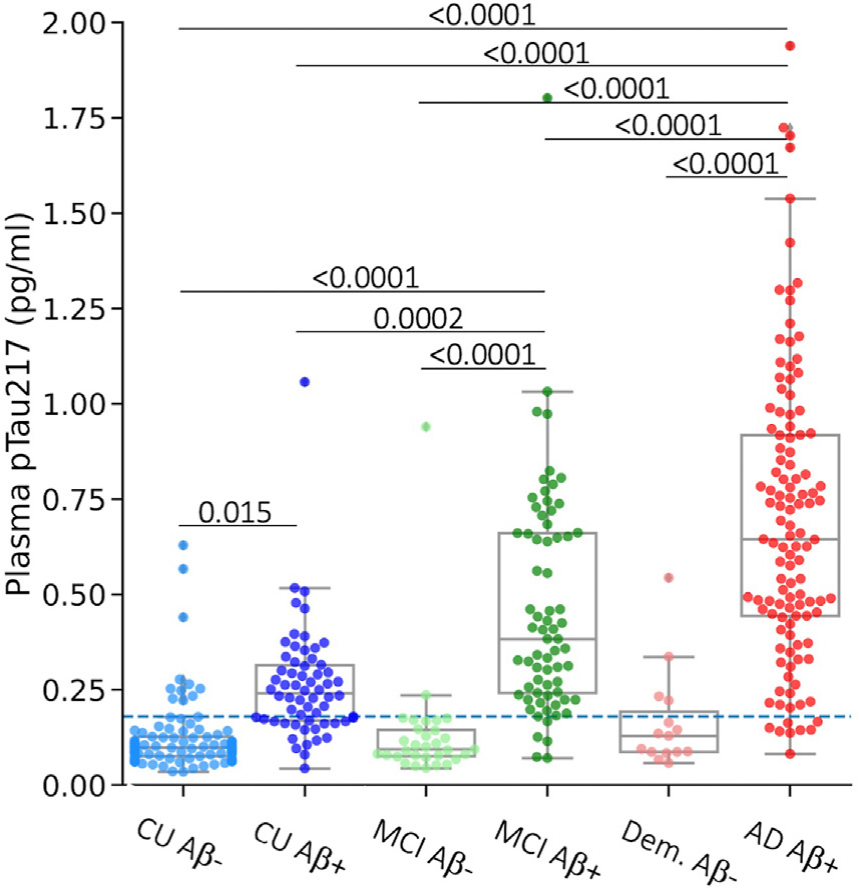
Plasma pTau217 concentration by clinical groups (CU, cognitively unimpaired, n = 156; MCI, mild cognitive impairment, n = 100; Dem., dementia, AD, Alzheimer's disease, n = 132 Dem + AD) stratified by Aβ PET status where Aβ+ means > 25 Centiloid. The error bars in the boxplots represent 1.5 times the interquartile range (IQR) above and below the upper (75th percentile) and lower (25th percentile) quartiles. The definition for outliers is when the data falls below Q1–1.5 × IQR or exceeds Q3 + 1.5 × IQR; Q1: the 25th percentile; Q3: the 75th percentile; IQR = Q3–Q1. Adjusted *p* are reported, corrected for multiple comparisons (Tukey HSD with family-wise error rate (FWER) < 0.05). The horizontal dashed line is the threshold for Aβ+ derived by Youden's index.

Fig. 1 shows the pTau217 levels in different clinical groups stratified by Aβ PET status. Concentrations are listed in the Supplementary Material. The median pTau217 concentration was significantly higher in Aβ+ MCI and Aβ+ AD relative to Aβ+ CU (adjusted *p* < 0.0001, Tukey HSD) and in Aβ+ AD relative to Aβ+ MCI (adjusted *p* < 0.0001, Tukey HSD). There was no significant difference between pTau217 levels in Aβ− CU (median (IQR): 0.1 (0.1–0.1) pg/ml), Aβ− MCI (median (IQR): 0.1 (0.1–0.1) pg/ml) and Aβ− Dementia (median (IQR): 0.1 (0.1–0.2) pg/ml).

### Correlation of plasma pTau217 with Aβ and tau PET

The correlation between plasma pTau217 and Aβ PET Centiloid was stronger within the entire cohort than within the CU and CI groups individually. In the whole cohort, the correlation demonstrated a Spearman's *r* of 0.76 [0.72–0.78] (adjusted *p* < 0.0001), while within the CU group, *r* was 0.64 [0.55–0.72] (adjusted *p* < 0.0001), and within the CI group, it was 0.60 [0.52–0.67] (adjusted *p* < 0.0001).

Assessment of the correlation between tau PET SUVR and plasma pTau217 in three ROIs demonstrated the strongest correlation within the MetaT ROI. The Spearman's correlation with MetaT tau SUVR was 0.78 [0.74–0.82] (adjusted *p* < 0.0001) within the whole cohort, *r =* 0.42 [0.29–0.53] (adjusted *p* < 0.0001) within the CU group, and *r* = 0.80 [0.76–0.84] (adjusted *p* < 0.0001) within the CI group. The correlations with Me and Te tau SUVR are listed in the Supplementary Material.

### Prediction of ^18^F-NAV4694 Aβ PET status by plasma pTau217

#### Area under curve

**Fig. 2:**
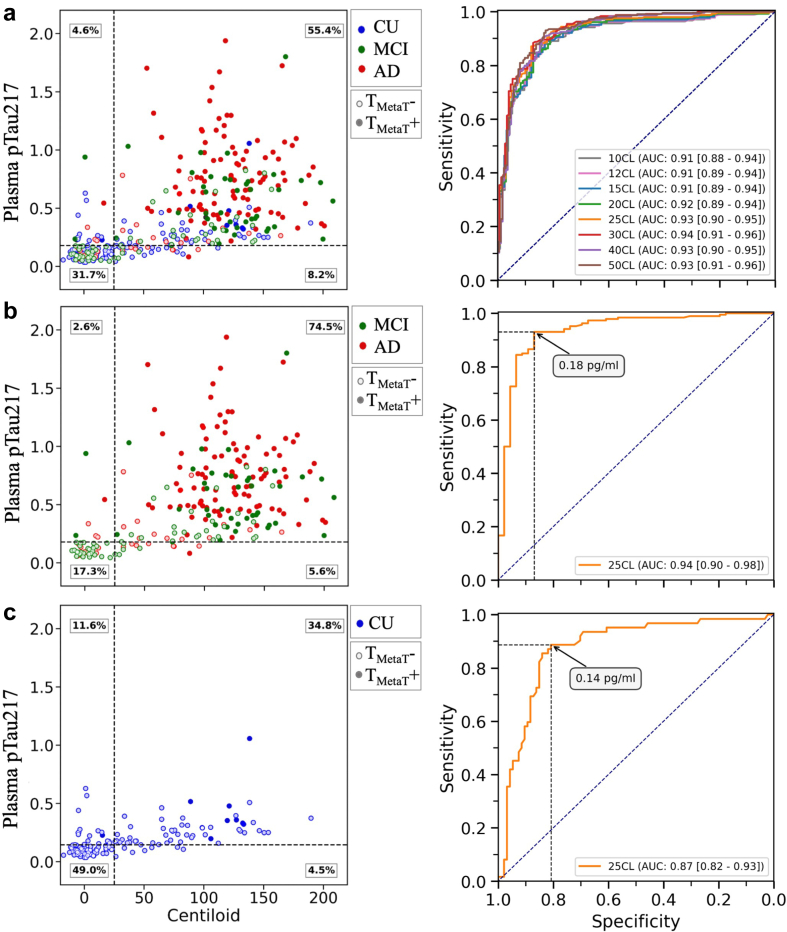
Receiver Operating Characteristic (ROC) curves and scatter plots for plasma pTau217 concentration vs Centiloid, for (**a)** the entire cohort (n = 388). (**b)** cognitively impaired participants (MCI or AD, n = 232). (**c)** cognitively unimpaired participants (n = 156). AUCs are reported with bootstrapped 95% confidence intervals shown in square brackets. The clinical groups are colour-coded: red for dementia, green for MCI, and blue for CU. Solid circles represent tau PET positive cases (in meta temporal region of interest). The ROC curves for the entire cohort were plotted using different Centiloid thresholds, ranging from 10 to 50, to define Aβ+ PET. In the scatter plots, the horizontal dashed line represents the pTau217 threshold derived from the Youden's index in each specific sub-cohort, and the vertical dashed line is the Centiloid threshold of 25 CL. In the ROC plots, the black dashed lines represent the sensitivity and specificity values corresponding to the annotated Youden threshold. CU, cognitively unimpaired; MCI, mild cognitive impairment; AD, Alzheimer's disease; T_MetaT_, tau in the meta temporal region of interest; CL, Centiloid; AUC, Area Under Curve.

When tested in the whole cohort, plasma pTau217 discriminated PET Aβ+ from PET Aβ−, with an AUC of 0.93 [0.90–0.95] (Fig. 2a). Among CI participants, pTau217 discriminated Aβ status with an AUC of 0.94 [0.90–0.98] (Fig. 2b). Among CU participants, the AUC was 0.87 [0.82–0.93] (Fig. 2c).

#### Applying Youden's index for binary classification into PET Aβ− or Aβ+

In the whole cohort, the Youden's index yielded a threshold concentration of 0.18 [0.14–0.18] pg/ml, yielding a sensitivity of 0.87 [0.85–0.95], specificity of 0.88 [0.79–0.92], PPV of 0.93 [0.88–0.95], and NPV of 0.79 [0.75–0.90]. The overall accuracy was 87% [85%–91%]. For a breakdown by CU and cognitively impaired (CI: MCI combined with dementia), see Supplementary Material.

#### Applying a two-threshold approach to classify to low, indeterminate, and high zones for Aβ PET positivity

**Fig. 3:**
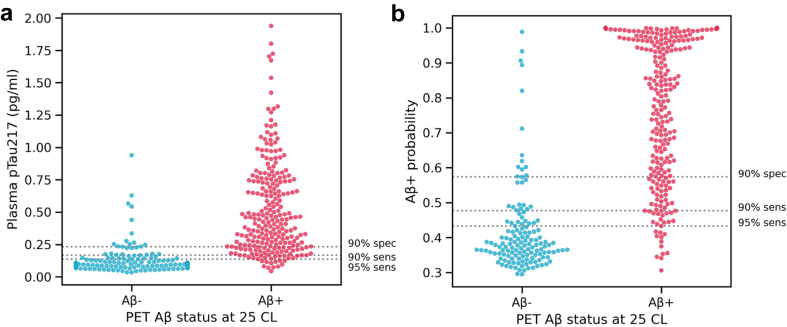
Applying a two-threshold approach to classify participants into Low, Indeterminate, and High zones for Aβ PET positivity (n = 388, of which 140 were Aβ− and 248 were Aβ+). **(a)** Plasma pTau217 concentration vs Aβ PET status (+/−). Horizontal dotted lines represent the lower thresholds of 0.17 pg/ml (90% sensitivity) and 0.14 pg/ml (95% sensitivity), and the upper threshold of 0.23 pg/ml (90% specificity). **(b)** Probability of Aβ PET positivity (derived from a logistic regression model including plasma pTau217 concentration) vs Aβ PET status. Using 90% sensitivity and 90% specificity thresholds, the Indeterminate zone included 11.3% of participants, and the overall correct classification for Low and High zones was 89%. Using 95% sensitivity and 90% specificity thresholds, the Indeterminate zone included 17.8% of participants, and the overall correct classification for Low and High zones was 92%. Sens, sensitivity; Spec, specificity.

To further optimise performance of pTau217, we employed a two-threshold approach to categorise participants to Low, Indeterminate and High zones (Fig. 3a and b). The optimal results were obtained when sensitivity was set to 95% to determine the lower threshold (0.14 pg/ml) and specificity was set to 90% to determine the upper threshold (0.23 pg/ml). Using this thresholding strategy, 52.6% of the entire cohort were classified into the High zone (>0.23 pg/ml), 29.6% classified into the Low zone (<0.14 pg/ml), with 17.8% remaining in the Indeterminate zone (between 0.14 and 0.23 pg/ml). Among those classified into the High zone, 94% were correctly classified as PET Aβ+. Among those classified into the Low zone, 90% were correctly classified as PET Aβ−. Overall, 92% of those in the Low or High zones were correctly classified.

### Prediction of ^18^F-MK6240 tau PET status by plasma pTau217

**Fig. 4:**
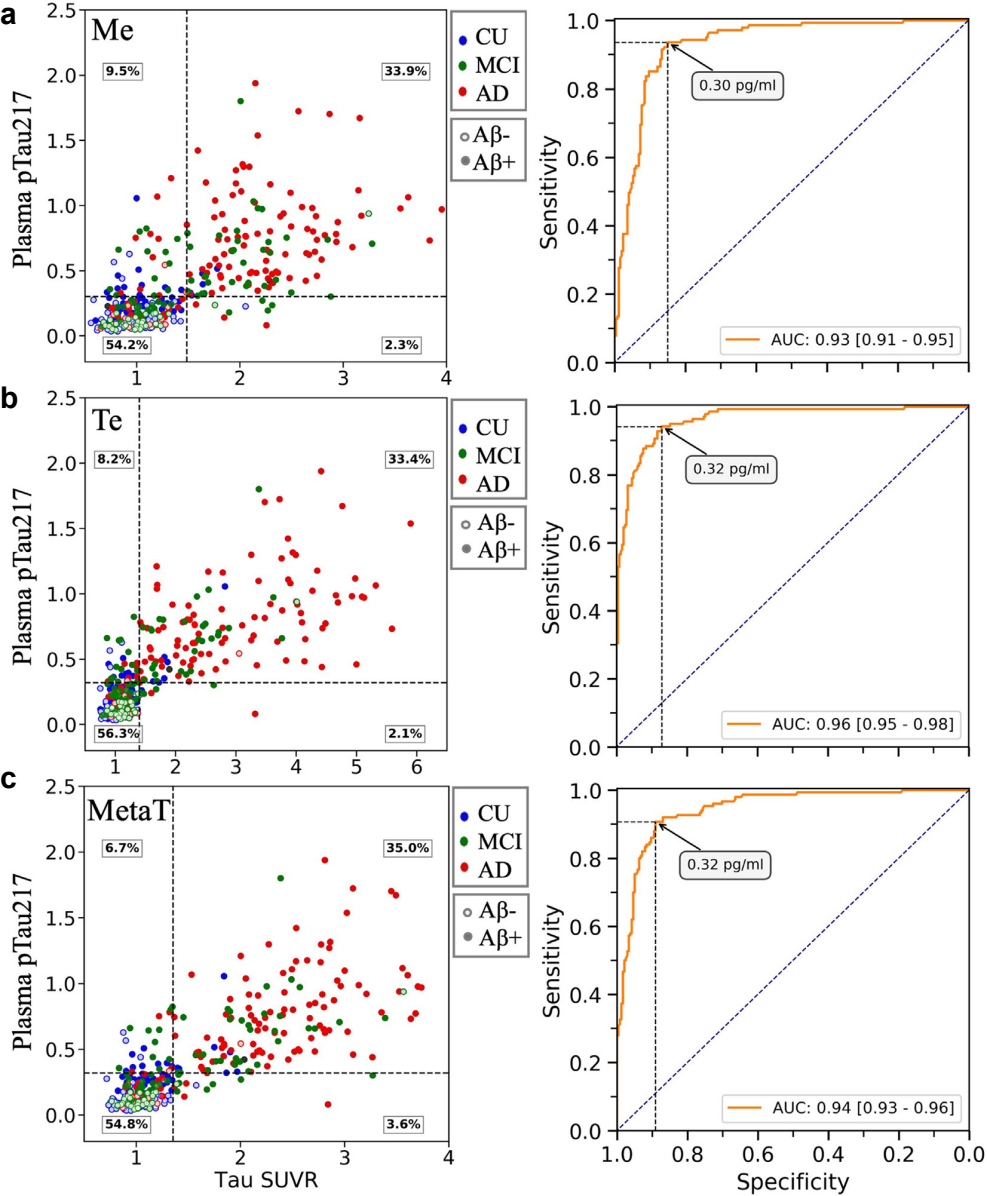
Receiver Operating Characteristic (ROC) curves and scatter plots of plasma pTau217 concentration vs tau PET SUVR in three regions of interest: **(a)** Me, **(b)** Te, and **(c)** MetaT, in the entire cohort (n = 388). AUCs are reported with bootstrapped 95% confidence intervals in square brackets. The clinical groups are colour-coded: red for dementia (n = 132), green for MCI (n = 100), and blue for CU (n = 156). Solid circles represent Aβ PET positive cases (>25 Centiloid). In the scatter plots, the horizontal dashed line represents the pTau217 threshold derived from the Youden's index. The vertical dashed line is the tau SUVR threshold, derived from mean + 2.5 SD of SUVR values in Aβ negative CU participants. In the ROC plots, the vertical and horizontal dashed lines represent the sensitivity and specificity values corresponding to the annotated Youden threshold. CU, cognitively unimpaired; MCI, mild cognitive impairment; AD, Alzheimer's disease; Me, mesial temporal; Te, temporoparietal; MetaT, meta temporal region of interest; SD, standard deviation; AUC, Area Under Curve.

The tau PET status was determined using a threshold derived from the 2.5 SD of the mean SUVR in the Aβ− CU group. Based on this definition of tau PET status, within the entire cohort, plasma pTau217 exhibited an AUC of 0.93 [0.91–0.95] for discriminating between tau+ and tau− in the Me ROI (Fig. 4a), AUC of 0.96 [0.95–0.98] for the Te ROI (Fig. 4b), and AUC of 0.94 [0.93–0.96] for the MetaT ROI (Fig. 4c). To discriminate between tau+ and tau-in the Me ROI, Youden threshold was 0.30 [0.30–0.37] pg/ml. This cut-off point gave a sensitivity of 0.94 [0.88–0.97], specificity of 0.85 [0.82–0.92], PPV of 0.78 [0.74–0.86] and NPV of 0.96 [0.93–0.98]. For discriminating between tau+ and tau− in the Te ROI, the Youden threshold of 0.32 [0.32–0.39] pg/ml provided a sensitivity of 0.94 [0.88–0.97], specificity of 0.87 [0.85–0.94], PPV of 0.80 [0.76–0.89], NPV of 0.96 [0.93–0.98]. For discriminating between tau+ and tau− in the MetaT ROI, the Youden threshold was the same: 0.32 [0.30–0.34] pg/ml. This threshold yielded sensitivity of 0.91 [0.87–0.95], specificity of 0.89 [0.85–0.93], PPV of 0.84 [0.78–0.89] and NPV of 0.94 [0.91–0.97]. For a breakdown by CU and CI, see Supplementary Table S3.

The Supplementary Figure S1 presents the results, wherein the determination of tau PET status was based on a threshold derived from the 95th percentile of the SUVR in the Aβ− CU group. This gave slightly lower AUC results, but all ROI AUC were >0.90 for the whole cohort analysis.

### Changes in pTau217 with advancing PET-based Braak and biological stages

**Fig. 5:**
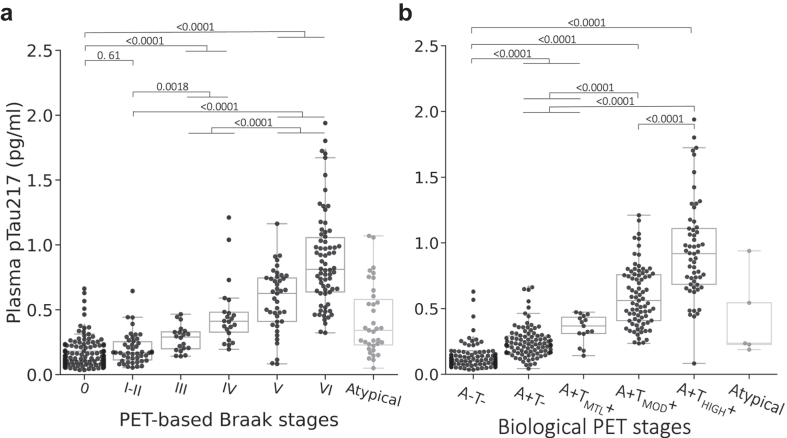
Changes in plasma pTau217 concentration across disease severity stages based on **(a)** PET-based Braak stages and **(b)** Revised Criteria for Diagnosis and Staging of Alzheimer's Disease: Alzheimer's Association Workgroup (2024). The error bars represent 1.5 times the interquartile range (IQR) above and below the upper (75th percentile) and lower (25th percentile) quartiles. The definition for outliers is when the data falls below Q1–1.5 × IQR or exceeds Q3 + 1.5 × IQR; Q1: the 25th percentile; Q3: the 75th percentile; IQR = Q3–Q1. Braak stages were grouped as I–II, III–IV, and V–VI for statistical comparisons. A−T−: Aβ negative & tau negative (n = 136); A+T−: Aβ positive & tau negative (n = 96); A+T_MTL_+: Aβ positive & tau uptake limited to medial temporal region (n = 15); A+T_MOD_+: Aβ positive & moderate tau uptake in temporo-parietal region (n = 79); A+T_HIGH_+: Aβ positive & high tau uptake in temporo-parietal region (n = 57); Atypical: participants with atypical patterns that did not follow the Braak stages (n = 35), or participants that could not be categorised to any of the biological PET stages (n = 5); adjusted *p* values were reported, corrected for multiple comparisons (Tukey HSD with family-wise error rate (FWER) < 0.05).

In the PET-based Braak tau staging, only a small number of participants (n = 3) were Braak stage II and therefore were grouped with stage I. Examining plasma pTau217 levels revealed an incremental trend across PET-based Braak stages (Fig. 5a). The pTau217 median (IQR) concentrations were 0.1 (0.1–0.2) pg/ml for the tau negative group (Braak 0), 0.2 (0.1–0.3) pg/ml for Braak I-II, 0.3 (0.2–0.3) pg/ml for Braak III, 0.4 (0.3–0.5) pg/ml for Braak IV, 0.6 (0.4–0.7) pg/ml for Braak V and 0.8 (0.6–1.1) pg/ml for Braak VI. Pairwise comparison of Braak 0, I–II, III–IV, and V–VI groups yielded significant differences between all except for Braak 0 and I–II (see Fig. 5a). Those that did not follow the order of the Braak staging scheme (n = 35) were categorised as ‘Atypical’, and their biomarker results are provided in the Supplementary Table S4.

Applying the biological PET staging, plasma pTau217 levels revealed a progressive trend across the stages (Fig. 5b). The pTau217 median (IQR) concentrations were 0.1 (0.1–0.1) pg/ml in the A−T− group, 0.2 (0.2–0.3) pg/ml in A+T−, 0.4 (0.3–0.4) pg/ml in A+T_MTL_+, 0.6 (0.4–0.8) pg/ml in A+T_MOD_+ and 0.9 (0.7–1.1) pg/ml in A+T_HIGH_+. Although there were higher concentrations of pTau217 in the A+T_MTL_+ group (effect size relative to A+T− = 0.88), the small sample size meant that this group's pTau217 level did not significantly differ from A+T−. Therefore, it was combined with A+T−. Statistical comparison results are presented in Fig. 5b. Those participants not meeting the biological PET stage criteria (n = 5) were classified as ‘Atypical’, and their biomarker results are detailed in the Supplementary Table S5.

### Discrimination between disease stages

**Fig. 6:**
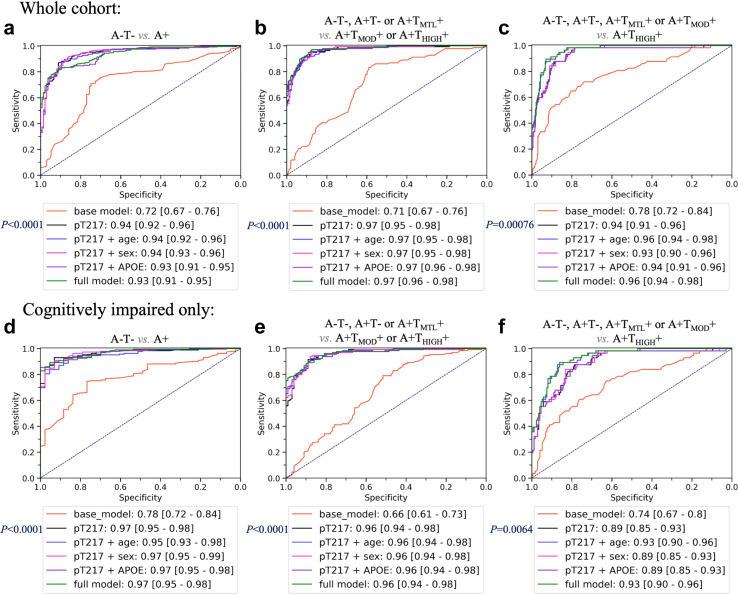
ROC analysis for biological PET staging **(a, d)** A−T− vs A+, **(b, e)** A−T−/A+T−/A+T_MTL_+ vs A+T_MOD_+/A+T_HIGH_+ and **(c, f)** A−T−/A+T−/A+T_MTL_+/A+T_MOD_+ vs A+T_HIGH_+. CU and CI combined (Top, n = 388), and CI only (Bottom, n = 232). Base model consists of age, sex, and Apolipoprotein E (*APOE*) ε4 status. Full model consists of pTau217, age, sex, and *APOE* ε4 status. AUC: area under the receiver operating characteristic curve with bootstrapped 95% confidence intervals in square brackets. A−T−: Aβ negative & tau negative; A+T−: Aβ positive & tau negative; A+T_MTL_+: Aβ positive & tau uptake limited to medial temporal region; A+T_MOD_+: Aβ positive & moderate tau uptake in temporo-parietal region; A+T_HIGH_+: Aβ positive & high tau uptake in temporo-parietal region. Adjusted *p* values were shown for comparison of the model with pTau217 only, to the base model (DeLong's test). Comparison of the model with pTau217 only to models with additional predictors (corrected for multiple comparisons) did not yield significant differences (DeLong's test).

The outcomes of ROC analyses for several models, utilizing plasma pTau217 alone or in combination with other predictors to distinguish between the biological PET stages, are illustrated in Fig. 6.

In the whole cohort (Fig. 6a–c), pTau217 outperformed the base model of age, sex and *APOE* ε4 when discriminating A+ vs A−T− (AUC = 0.94 [0.92–0.96] vs 0.72 [0.67–0.76]; adjusted *p* < 0.0001, DeLong's test), A+T_MOD_+/A+T_HIGH_+ vs A−T−/A+T−/A+T_MTL_+ (AUC = 0.97 [0.95–0.98] vs 0.71 [0.67–0.76]; adjusted *p* < 0.0001, DeLong's test), and A+T_HIGH_+ vs A−T−/A+T−/A+T_MTL_+/A+T_MOD_+ (AUC = 0.94 [0.91–0.96] vs 0.78 [0.72–0.84]; adjusted *p* = 0.00076, DeLong's test). Age, sex or *APOE* ε4 allele status did not improve the ability of pTau217 to discriminate between the stages. The Youden threshold-based sensitivity, specificity, NPV, PPV and accuracy parameters, for the models including pTau217 only, are provided in Supplementary Table S6.

**Fig. 7:**
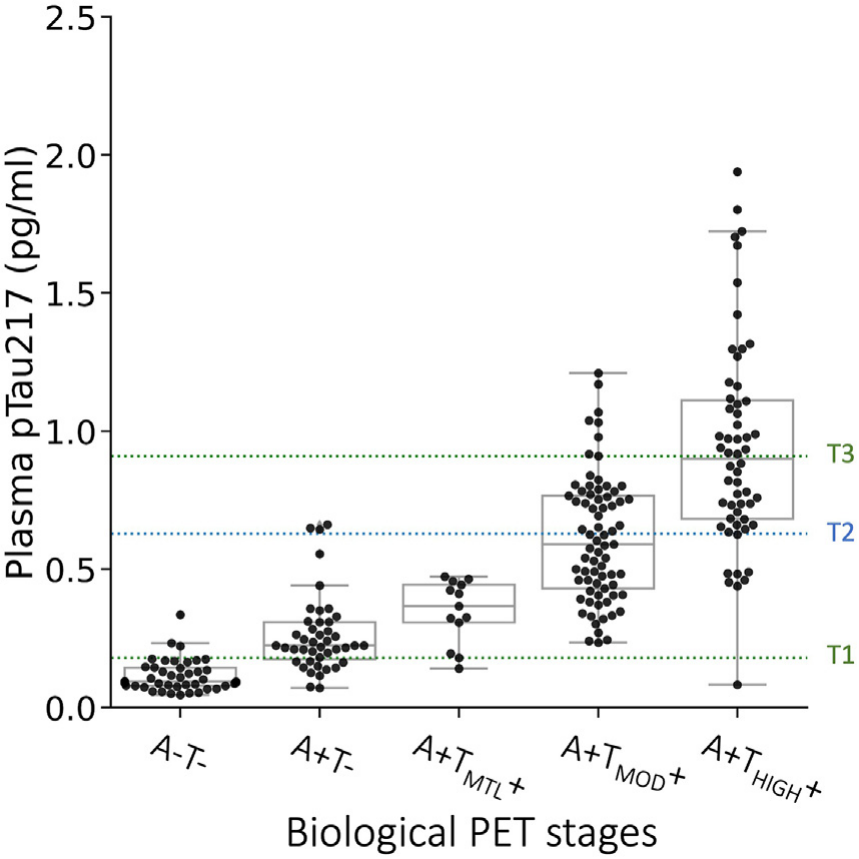
Cognitively impaired cohort plasma pTau217 thresholds for AD biological stage. T1: Youden threshold in CI for discrimination between A−T− and all A+ (0.18 pg/ml). T2: Youden threshold in CI for discrimination of A−T−/A+T−/A+T_MTL_+/A+T_MOD_+ from A+T_HIGH_+ (0.63 pg/ml). T3: 95% specificity threshold for A+T_HIGH_+ (0.91 pg/ml). The error bars represent 1.5 times the interquartile range (IQR) above and below the upper (75th percentile) and lower (25th percentile) quartiles. The definition for outliers is when the data falls below Q1–1.5 × IQR or exceeds Q3 + 1.5 × IQR; Q1: the 25th percentile; Q3: the 75th percentile; IQR = Q3–Q1; A−T−: Aβ negative & tau negative (n = 43); A+T−: Aβ positive & tau negative (n = 43); A+T_MTL_+: Aβ positive & tau uptake limited to medial temporal region (n = 13); A+T_MOD_+: Aβ positive & moderate tau uptake in temporo-parietal region (n = 73); A+T_HIGH_+: Aβ positive & high tau uptake defined as in the top 25% of tau values in Aβ positive CI individuals in the temporo-parietal region (n = 56).

In the CI group (Fig. 6d–f), the model with pTau217 outperformed the base model, for discrimination of A+ vs A−T− (AUC = 0.97 [0.95–0.98] vs 0.78 [0.72–0.84]; adjusted *p* < 0.0001, DeLong's test), A+T_MOD_+/A+T_HIGH_+ vs A−T−/A+T−/A+T_MTL_+ (AUC = 0.96 [0.94–0.98] vs 0.66 [0.61–0.73]; adjusted *p* < 0.0001, DeLong's test) and A+T_HIGH_+ vs A−T−/A+T−/A+T_MTL_+/A+T_MOD_+ (AUC = 0.89 [0.85–0.93] vs 0.74 [0.67–0.8]; adjusted *p* = 0.0064, DeLong's test). Age, sex or *APOE* ε4 allele status did not improve the ability of pTau217 to discriminate between the stages. The Youden threshold-based sensitivity, specificity, NPV, PPV and accuracy parameters for the models with only pTau217 are provided in the Supplementary Table S7. A higher pTau217 threshold at 95% specificity for A+T_HIGH_+ was examined (0.91 pg/ml), as shown in Fig. 7, identifying 50% of those with high tau but there was some overlap with the Moderate PET tau group above this threshold.

ROC analyses for plasma pTau217 to distinguish between the participants with lower Braak stages (0–III) and those with higher Braak stages (IV–VI) gave an AUC of 0.96 [0.94–0.98] for the whole cohort with similar result in the CI subgroup and are detailed in Supplementary Figure S2.

## Discussion

This study presents the findings for a fully automated Lumipulse G pTau217 plasma assay against the latest generation Aβ and tau PET tracers for AD. Importantly, concentrations determined with the Lumipulse G pTau217 plasma appear to be specific to AD pathology, in that it only rose in Aβ+ participants, and its levels exhibited a ∼two-fold increase in the Aβ+ CU, four-fold increase in the Aβ+ MCI and a six-fold increase in the Aβ+ dementia, relative to the Aβ− CU group. Our findings also showcased the robust discriminative performance of pTau217 for negative vs positive Aβ PET status (based on various CL thresholds; AUC range: 0.91–0.94), negative vs positive tau PET status (in three different ROIs; AUC range: 0.93–0.96), and for identifying Aβ+ individuals with moderate/high neocortical tau (AUC: 0.97) or high neocortical tau (AUC: 0.94).

Various studies have investigated if pTau biomarkers are more associated with the presence of cerebral Aβ or with tau aggregation. Stronger association of plasma pTau231, pTau181 and pTau217 levels with brain Aβ-pathology than tau PET has been reported.^7^^,^^20^, ^21^, ^22^ These findings have been interpreted to suggest that the release of pTau might be an early physiological reaction to brain Aβ plaques, occurring prior to the widespread aggregation of neurofibrillary tangles. While we observed this relationship in CU, the pTau217 levels in CI were, in contrast, more related to aggregated tau (*r* = 0.8) than to Aβ (*r* = 0.6) pathology on PET to the point that it enabled staging of AD based on brain tau levels. This stronger association with tau PET in CI is consistent with the observation from Aβ monoclonal antibody trials that the reduction in plasma pTau is much less than the substantial reduction achieved in Aβ levels on PET while tau PET showed limited change.^1^

The Lumipulse G pTau217 plasma assay revealed excellent performance, with almost all AUCs larger than 0.90 for PET Aβ+ vs Aβ−, using various abnormality thresholds, and for PET tau+ vs tau−, in different ROIs. When comparing these results to those reported for other pTau immunoassays (AUC range: 0.64–0.89),^2^^,^^4^ Lumipulse pTau217 demonstrated a favourable performance, akin to what has been previously reported for mass spectrometry-based p-Tau217 (p-Tau217^WashU^: AUC = 0.95).^4^ However, given the variations arising from the likelihood of differences in cohort demographics, disease prevalence and clinical symptoms, only head-to-head studies will allow direct comparison of performance of the different platforms/tests.

We evaluated the accuracy of pTau217 in classifying participants based on their Aβ PET status using two methods. The first involved the conventional use of Youden's index threshold for binary classification. The second method employed a two-threshold approach, categorizing participants into three zones for Aβ PET positivity: Low, Indeterminate, and High. For the latter approach, pTau217 thresholds set to 95% sensitivity and 90% specificity were selected, as they yielded a minimum of 90% agreement between pTau217 status and Aβ PET status in the Low and High zones, with the smallest indeterminate zone. Applying pTau217 thresholds set to 95% sensitivity and 95% specificity would have yielded 94% correct classification for the combined Low and High zones which is consistent with the performance reported for the ALZpath pTau217 Simoa based assay.^8^

Our data also suggested good performance for the Lumipulse G pTau217 plasma assay for disease staging. We showed significant increments in plasma levels of pTau217 with disease severity as determined by both PET-based Braak stages (eight-fold increase from Braak 0 to Braak VI) and biological PET stages (nine-fold increase from A−T− to A+T_HIGH_+). This aligns with earlier studies documenting a 300–700% rise in plasma levels of pTau217 among individuals with symptomatic AD.^2^^,^^23^

A crucial insight gained from a recent successful therapeutic AD trial indicated that individuals with ‘low/medium’ brain tau levels derived greater benefit from Aβ monoclonal antibody treatment compared to those with a ‘high’ brain tau burden defined as the upper quartile of Aβ+ MCI/AD individuals on tau PET.^1^ Consequently, it becomes imperative to ascertain whether plasma biomarkers can serve not only in selecting patients with high Aβ for therapy but also in distinguishing different AD biological PET stages (i.e., different levels of tau burden). This distinction is pivotal for more accurately predicting the potential benefits of treatment. Our data showed that the Lumipulse G pTau217 plasma assay could be used for distinguishing individuals at Intermediate (A+T_MOD_+) or Advanced (A+T_HIGH_+) stages of AD vs those at lower stage or no AD with an AUC of 0.97, potentially aiding in their inclusion in or exclusion from a therapeutic trial or intervention. While the assay had excellent NPV for Advanced (A+T_HIGH_+) stage AD in the overall cohort and in the CI cohort (0.95 [0.92–1.0]), due to notable overlap between A+T_MOD_+ and A+T_HIGH_+ plasma pTau217 levels, the PPV was poor (0.57 [0.44–0.69]). At a 95% specificity threshold only 50% of Advanced (A+T_HIGH_+) stage AD were identified. Consistent with our findings, a study using the Lilly pTau217 Meso Scale Discovery assay reported an AUC of approximately 0.90, discriminating Aβ+ individuals with high tau load from all others. However they also found substantial overlap in the pTau217 levels between those with ‘intermediate’ and ‘high’ PET tau so used the strong NPV to model cost savings from screening out those below a plasma pTau217 threshold with high NPV and then used tau PET to identify high tau in those above the threshold.^24^

Study limitations included unmeasured confounding factors in our analyses and the lack of validation of the optimal pTau217 thresholds derived from our cohort in an independent cohort. Disease prevalence has substantial impact on positive and negative predictive values. In the current cohort, the prevalence of Aβ+ PET (>25CL) was relatively high, at 40% in CU, 69% in MCI, and 89% in participants with dementia, compared to many cohorts. Furthermore, the participants were drawn from AIBL and ADNeT research cohorts, and our findings may not be generalizable to “real world” settings such as patients referred to memory clinics or primary care or ethnically more diverse populations, where not only disease prevalence may differ, but also the extent of medical comorbidities such as renal impairment that may affect pTau levels: future research would benefit from assessing the performance of the Lumipulse G pTau217 plasma assay in such settings.

In conclusion, our evaluation of the Lumipulse G pTau217 plasma assay demonstrates high accuracy for detecting AD pathology in symptomatic individuals and exclusion of AD pathology in asymptomatic persons, and a strong correlation with tau PET that assists staging of AD, on a widely available platform that will facilitate clinical implementation.

## Contributors

AF, JDD, VD, CCR contributed to the study concept and design, formal analysis of data, visualisation, and writing the original draft. VLV contributed to methodology. VD and JDD contributed to validation. PB, VD and KH contributed to data curation. LW, CCR, CLM, CF, RNM contributed to funding acquisition. VLV, PB, SML, NK, KH, CF, JR, LM, SA, RNM, SRRS, KT, ES, AWB, CLM, JF contributed to review & editing. AF and JDD have verified the underlying data reported in the manuscript. All authors contributed to critical review of the manuscript, provided final approval of the version to be published and accept responsibility for the accuracy and integrity of the reported work.

## Data sharing statement

The underlying data reported in the manuscript are available from the corresponding author upon reasonable request. Access to the deidentified data can be achieved by submitting an expression of interest (EOI) to AIBL via www.aibl.csiro.au and ADNeT via https://www.australiandementianetwork.org.au/researcher/expression-of-interest.

## Declaration of interests

CCR has received research grants from NHMRC, Enigma Australia, Biogen, Eisai and Abbvie. He is on the scientific advisory board for Cerveau Technologies and has consulted for Prothena, Eisai, Roche, and Biogen Australia. VLV has received a grant from NIA and is and has been a consultant or paid speaker at sponsored conference sessions for Eli Lilly, Life Molecular Imaging, ACE Barcelona, and BRI Japan. ES and AWB are employees of Abbvie. SML is a scientific advisor for Cytox Ltd. SA has received grants from NHMRC, MRFF, and NIH and consulting fees from Eisai Australia. SRS has received grants from NHMRC, Alzheimer's Association (USA), Alzheimer's Drug Discovery Foundation, and Bright Focus Foundation and had a paid role in MRFF Grant Assessment Committee. The other authors did not report any conflict of interest.

## Figures and Tables

**Supplementary Figure S2:**