# Supplementary material for: Detection and staging of Alzheimer's disease by plasma pTau217 on a high throughput immunoassay platform
Source: eBioMedicine. 2024 Oct 21;109:105405. doi: 10.1016/j.ebiom.2024.105405 (PMC11536028; doi:10.1016/j.ebiom.2024.105405)
Supplement: Supplementary material [file mmc1.docx]

**SUPPLEMENTARY MATERIAL**

Detection and staging of Alzheimer’s disease by plasma pTau217 on a high throughput immunoassay platform

**TABLE OF CONTENTS**

**RESULTS**

- Plasma pTau217 concentration in clinical groups by Aβ status
- Correlation of plasma pTau217 with Me and Te tau PET
- Prediction of ^18^F-NAV4694 Aβ PET status by plasma pTau217 (CI and CU)
- Prediction of ^18^F-MK6240 tau PET status by plasma pTau217 (CI and CU)
- Supplementary Table 1
- Supplementary Table 2
- Supplementary Table 3
- Supplementary Table 4
- Supplementary Table 5
- Supplementary Table 6
- Supplementary Table 7
- Supplementary Figure 1
- Supplementary Figure 2

**RESULTS**

**Plasma pTau217 concentration in clinical groups by Aβ status**

The median (IQR) concentration of pTau217 was 0.1 (0.1-0.1) pg/ml in the Aβ- CU, while it showed a 2-fold increase in the Aβ+ CU (0.2 (0.2-0.3) pg/ml, adjusted *p* = 0.015), a 4-fold increase in the Aβ+ MCI (0.4 (0.2-0.7) pg/ml, adjusted *p* < 0.0001) and a 6-fold increase in Aβ+ AD (0.6 (0.4-0.9) pg/ml, adjusted *p* < 0.0001).

**Correlation of plasma pTau217 with Me and Te tau PET**

The correlation with Me tau SUVR had a Spearman’s *r* of 0.72 ﻿[0.68 - 0.76] (adjusted *p* <0.0001) within the whole cohort, *r =* 0.37 ﻿[0.24 - 0.49] (adjusted *p* <0.0001) within the CU group, and *r* = 0.68 ﻿[0.62 - 0.74] (adjusted *p* <0.0001) within the CI group. The correlation with Te tau SUVR exhibited a Spearman’s *r* value of 0.71 ﻿[0.66 - 0.77] (adjusted *p* <0.0001) in the whole cohort, *r =* 0.25 ﻿[0.12 - 0.38] (adjusted *p* = 0.019) in the CU group, and *r* = 0.79 ﻿[0.74 - 0.82] (adjusted *p* <0.0001) in the CI group.

**Prediction of ^18^F-NAV4694 Aβ PET status by plasma pTau217 (CI and CU)**

*Applying Youden’s index for binary classification into* *PET Aβ- or Aβ+:*

In the CI group, the Youden’s index threshold was the same as for the entire cohort: 0.18 [0.18 - 0.24] pg/ml which provided sensitivity of 0.93 [0.83 - 0.96], specificity of 0.87 [0.82 - 1.00], PPV of 0.97 [0.95 – 1.00], and NPV of 0.75 [0.56 - 0.86].

In the CU, Youden’s index threshold of 0.14 [0.14 - 0.16] pg/ml gave a sensitivity of 0.89 [0.81 - 0.96], specificity of 0.81 [0.76 - 0.90], PPV of 0.75 [0.69 - 0.86] and NPV of 0.92 [0.86 - 0.97].

**Prediction of ^18^F-MK6240 tau PET status by plasma pTau217 (CI and CU)**

In the CI group (Supplementary Table 3), an AUC value of 0.95 [0.93 - 0.97] was observed in the Te ROI, with the Youden threshold of 0.37 [0.33 - 0.46] pg/ml which was higher than the one observed for the whole cohort (0.32 pg/ml). Threshold of 0.37 pg/ml provided sensitivity of 0.92 [0.85 - 0.97], specificity of 0.88 [0.82 - 0.95], PPV of 0.91 [0.86 - 0.95], and NPV of 0.89 [0.83 - 0.96].

In the CU group (Supplementary Table 3), both Me and Te ROIs had an AUC of 0.91 (Me: 0.91 [0.86 - 0.96] and Te: 0.91 [0.85 - 0.97]) while MetaT ROI had an AUC value of 0.90 [0.84 - 0.96] and the Youden threshold ranged between 0.20 to 0.31 pg/ml.

**Supplementary Table 1. Demographic characteristics and statistical comparisons, breakdown by clinical groups**

|  | **CU** | **MCI** | **Dementia** | ***p* _all_** | ***p* _MCI vs. CU_** | ***p* _Dementia vs. CU_** |
| --- | --- | --- | --- | --- | --- | --- |
| ***Sample size*** | 156 | 100 | 132 | .. |  |  |
| ***Age (years), mean* ± *SD*** | 75.3 ± 5.6 | 73.7 ± 7.8 | 70.2 ± 7.7 | <0.0001 | 0.17 | <0.0001 |
| ***Education (years), median (IQR)*** | 15.0 (11.0-16.0) | 12.0 (10.0-15.0) | 11.0 (10.0-15.0) | <0.0001 | 0.0014 | <0.0001 |
| ***Sex, male (%)*** | 49 | 56 | 52 | 0.58 | .. | .. |
| ***APOE ε4+ (%)*** | 37 | 59 | 67 | <0.0001 | 0.0022 | <0.0001 |
| ***MMSE, median (IQR)*** | 29.0 (28.0-30.0) | 27.0 (25.0-28.0) | 23.0 (21.0-24.0) | <0.0001 | <0.0001 | <0.0001 |
| ***CDR SoB, median (IQR)*** | 0.0 (0.0-0.0) | 1.0 (0.5-2.0) | 4.0 (3.5-5.0) | <0.0001 | <0.0001 | <0.0001 |
| ***Centiloid, median (IQR)*** | 11.9 (-1.9-66.6) | 89.6 (7.4-127.9) | 108.4 (76.0-136.1) | <0.0001 | <0.0001 | <0.0001 |
| ***MK6240 SUVR_MetaT_, median (IQR)*** | 1.0 (0.9-1.1) | 1.2 (1.0-1.9) | 2.1 (1.4-2.7) | <0.0001 | <0.0001 | <0.0001 |
| ***Plasma pTau217, pg/ml, median (IQR)*** | 0.1 (0.1-0.2) | 0.3 (0.1-0.6) | 0.6 (0.3-0.9) | <0.0001 | <0.0001 | <0.0001 |
| ***Aβ PET+ (%)*** | 40 | 69 | 89 | <0.0001 | <0.0001 | <0.0001 |
| ***MetaT Tau PET+ (%)*** | 6 | 41 | 76 | <0.0001 | <0.0001 | <0.0001 |

CU: cognitively unimpaired; MCI: mild cognitive impairment; SD: standard deviation; *APOE* ε4+: Apolipoprotein E ε4 positive; MMSE: mini-mental state examination; CDR-SoB: clinical dementia rating scale sum of boxes; SUVR_MetaT_: SUVR in the meta temporal region. Aβ PET+, based on a Centiloid threshold of 25 CL; Tau PET+, based on meta temporal region; *P*_all_ column indicates whether there were any statistically significant differences between the three groups (one-way ANOVA, Kruskal-Wallis test or chi-square (χ²) test). *p* _MCI vs. CU_ and *p* _Dementia vs. CU_ columns present pairwise comparisons (Tukey's HSD or chi-square (χ²) test).

**Supplementary Table 2. Demographic characteristics, breakdown by biological PET stages**

|  | **A-T-** | **A+T-** | **A+T_MTL_+** | **A+T_MOD_+** | **A+T_HIGH_+** |
| --- | --- | --- | --- | --- | --- |
| ***Sample size*** | 136 | 96 | 15 | 79 | 57 |
| ***Age (years), mean* ± *SD*** | 73.3 ± 6.2 | 75.5 ± 7.1 | 74.3 ± 5.0 | 74.3 ± 6.9 | 67.1 ± 8.0 |
| ***Education (years), median (IQR)*** | 15.0 (11.0-16.0) | 12.0 (10.0-15.0) | 11.0 (10.0-13.5) | 12.0 (10.0-15.0) | 11.0 (10.0-15.0) |
| ***Sex, male (%)*** | 52 | 55 | 53 | 53 | 46 |
| ***APOE ε4+ (%)*** | 24 | 56 | 100 | 80 | 67 |
| ***MMSE, median (IQR)*** | 28.0 (27.0-29.0) | 27.0 (25.8-29.0) | 26.0 (24.0-27.5) | 24.0 (22.0-27.0) | 23.0 (20.0-25.0) |
| ***CDR SoB, median (IQR)*** | 0.0 (0.0-0.5) | 0.5 (0.0-2.0) | 1.0 (0.5-3.0) | 3.5 (1.5-4.2) | 4.0 (3.5-5.0) |
| ***Centiloid, median (IQR)*** | 0 0.0 (-4.6-7.5) | 74.8 (43.3-106.1) | 113.0 (94.2-135.5) | 124.9 (97.4-147.1) | 120.0 (100.7-138.3) |
| ***MK6240 SUVR_MetaT_, median (IQR)*** | 1.0 (0.9-1.1) | 1.1 (0.9-1.2) | 1.5 (1.4-1.6) | 2.0 (1.7-2.2) | 2.8 (2.5-3.1) |
| ***Plasma pTau217, pg/ml, median (IQR)*** | 0.1 (0.1-0.1) | 0.2 (0.2-0.3) | 0.4 (0.3-0.4) | 0.6 (0.4-0.8) | 0.9 (0.7-1.1) |
| ***Aβ PET+ (%)*** | 0 | 100 | 100 | 100 | 100 |
| ***MetaT Tau PET+ (%)*** | 0 | 3 | 80 | 94 | 100 |

*A-T-* Aβ negative & tau negative; *A+T-* Aβ positive & tau negative; *A+T_MTL_+* Aβ positive & tau uptake limited to medial temporal region; *A+T_MOD_+* Aβ positive & moderate tau uptake in temporo-parietal region; *A+T_HIGH_+* Aβ positive & high tau uptake in temporo-parietal region. *APOE* ε4+: Apolipoprotein E ε4 positive; MMSE: mini-mental state examination; CDR-SoB: clinical dementia rating scale sum of boxes; SUVR_MetaT_: SUVR in the meta temporal region. Aβ PET+, based on a Centiloid threshold of 25 CL; Tau PET+, based on meta temporal region.

**Supplementary Table 3. ROC parameters for discriminating negative vs positive tau PET status using plasma pTau217, *in the CU and CI groups separately***

|  | **Me** | **Te** | **MetaT** |
| --- | --- | --- | --- |
| **CU** | | | |
| AUC | 0.91 [0.87 - 0.95] | 0.91 [0.87 - 0.96] | 0.90 [0.83 - 0.97] |
| Youden threshold | 0.31 [0.23 - 0.32] | 0.23 [0.23 - 0.32] | 0.20 [0.20 - 0.35] |
| Sensitivity | 0.86 [0.83 - 1.0] | 1.00 [0.86 - 1.0] | 1.00 [0.80 - 1.00] |
| Specificity | 0.91 [0.71 - 0.93] | 0.74 [0.71 - 0.93] | 0.71 [0.67 - 0.96] |
| PPV | 0.26 [0.08 - 0.36] | 0.16 [0.10 - 0.33] | 0.16 [0.08 - 0.50] |
| NPV | 0.99 [0.99 - 1.00] | 1.00 [0.99 - 1.00] | 1.00 [0.99 - 1.00] |
| **CI** | | | |
| AUC | 0.89 [0.85 - 0.93] | 0.95 [0.93 - 0.97] | 0.92 [0.89 - 0.95] |
| Youden threshold | 0.32 [0.30 - 0.38] | 0.37 [0.33 - 0.47] | 0.37 [0.30 - 0.38] |
| Sensitivity | 0.93 [0.85 - 0.96] | 0.92 [0.84 - 0.97] | 0.87 [0.84 - 0.95] |
| Specificity | 0.80 [0.75 - 0.90] | 0.88 [0.82 - 0.96] | 0.89 [0.80 - 0.94] |
| PPV | 0.86 [0.82 - 0.93] | 0.91 [0.86 - 0.96] | 0.92 [0.87 - 0.96] |
| NPV | 0.89 [0.80 - 0.94] | 0.89 [0.82 - 0.96] | 0.81 [0.77 - 0.92] |

Values are presented as mean [bootstrapped 95% confidence interval]. Tau PET status determined using a threshold derived from the mean + 2.5 standard deviations of the standardized uptake value ratio (SUVR) in the Aβ- CU group. CU: cognitively unimpaired. CI: cognitively impaired. AUC: area under the ROC curve. PPV: positive predictive value. NPV: negative predictive value. Me: mesial temporal region of interest, which comprised entorhinal cortex, hippocampus, parahippocampus and amygdala. Te: temporoparietal region of interest, consisting of inferior temporal, fusiform, supramarginal and angular gyri, posterior cingulate/precuneus, superior and inferior parietal, and lateral occipital cortex. MetaT: meta temporal region of interest, which consisted of the Me region as well as inferior and middle temporal and fusiform gyri.

**Supplementary Table 4. Biomarker results for the participants categorised as Braak Atypical.**

| **Centiloid (CL)** | **Braak1 (SUVR)** | **Braak2 (SUVR)** | **Braak3 (SUVR)** | **Braak4 (SUVR)** | **Braak5 (SUVR)** | **Braak6 (SUVR)** | **pTau217 (pg/ml)** |
| --- | --- | --- | --- | --- | --- | --- | --- |
| -11.40 | 1.65 | 0.87 | 0.99 | 0.99 | 1.07 | 1.05 | 0.124 |
| -7.93 | 1.64 | 1.00 | 1.12 | 0.96 | 0.96 | 1.00 | 0.146 |
| -2.55 | 1.94 | 0.98 | 1.11 | 0.92 | 0.90 | 0.83 | 0.113 |
| 3.99 | 1.17 | 0.89 | 1.07 | 1.06 | 1.04 | 1.09 | 0.05 |
| 10.89 | 1.54 | 0.98 | 1.11 | 0.94 | 0.90 | 0.90 | 0.222 |
| 16.58 | 1.46 | 0.89 | 1.47 | 1.72 | 1.60 | 1.14 | 0.544 |
| 27.62 | 2.03 | 0.95 | 1.10 | 0.84 | 0.83 | 0.88 | 0.234 |
| 32.22 | 1.17 | 0.92 | 1.11 | 0.99 | 0.98 | 0.99 | 0.783 |
| 32.50 | 1.15 | 0.84 | 1.10 | 0.94 | 0.93 | 0.96 | 0.167 |
| 43.06 | 1.01 | 1.03 | 1.07 | 1.01 | 1.02 | 0.92 | 0.15 |
| 43.90 | 1.21 | 0.96 | 1.14 | 1.01 | 0.92 | 0.89 | 0.358 |
| 57.57 | 1.44 | 0.96 | 1.10 | 0.96 | 0.91 | 0.89 | 0.556 |
| 68.62 | 1.33 | 0.96 | 1.13 | 0.96 | 0.89 | 0.86 | 0.649 |
| 73.38 | 1.27 | 0.90 | 1.05 | 0.98 | 1.06 | 1.02 | 0.188 |
| 73.88 | 1.16 | 0.78 | 1.29 | 0.93 | 1.00 | 1.29 | 0.262 |
| 83.04 | 1.59 | 0.94 | 1.19 | 0.96 | 0.94 | 1.02 | 0.263 |
| 89.05 | 1.17 | 0.65 | 1.14 | 0.89 | 0.95 | 0.86 | 0.753 |
| 95.17 | 1.41 | 0.90 | 1.13 | 0.94 | 0.88 | 0.91 | 0.237 |
| 105.84 | 1.68 | 0.96 | 1.21 | 0.94 | 0.92 | 0.84 | 0.198 |
| 119.61 | 1.45 | 1.00 | 1.18 | 1.24 | 1.07 | 0.91 | 0.353 |
| 124.91 | 1.33 | 0.74 | 1.24 | 1.09 | 1.25 | 1.19 | 0.604 |
| 126.74 | 1.87 | 0.99 | 1.15 | 0.92 | 0.86 | 0.88 | 0.396 |
| 127.17 | 1.65 | 0.99 | 1.26 | 0.97 | 0.90 | 0.90 | 0.359 |
| 132.34 | 1.84 | 1.01 | 1.55 | 1.28 | 1.00 | 0.85 | 0.331 |
| 133.94 | 1.72 | 0.96 | 1.18 | 0.93 | 0.93 | 1.00 | 0.248 |
| 135.21 | 1.39 | 0.81 | 1.06 | 1.19 | 1.14 | 0.90 | 0.824 |
| 138.18 | 1.12 | 0.68 | 1.47 | 1.48 | 1.47 | 1.10 | 1.057 |
| 145.88 | 2.50 | 1.37 | 2.21 | 1.23 | 1.01 | 1.32 | 0.492 |
| 146.33 | 1.66 | 0.89 | 1.16 | 0.89 | 0.89 | 0.94 | 0.266 |
| 149.73 | 1.27 | 0.84 | 1.16 | 1.19 | 1.31 | 1.02 | 0.746 |
| 150.82 | 1.24 | 0.65 | 1.14 | 1.08 | 1.14 | 1.11 | 0.802 |
| 153.88 | 1.48 | 0.99 | 1.20 | 0.96 | 0.89 | 0.90 | 0.251 |
| 159.34 | 1.75 | 0.99 | 1.71 | 0.95 | 0.81 | 0.95 | 0.341 |
| 167.80 | 1.42 | 0.83 | 1.33 | 1.20 | 1.15 | 1.10 | 1.069 |
| 185.14 | 1.96 | 1.00 | 1.99 | 1.43 | 1.27 | 1.63 | 0.541 |

SUVR: ^18^F-MK6240 tau standardized uptake value ratio.

**Supplementary Table 5. Biomarker results for the participants classified as ‘Atypical’ for biological PET staging**

| **Centiloid (CL)** | **MK6240 SUVR_Me_** | **MK6240 SUVR_Te_** | **MK6240 SUVR_R_** | **pTau217 (pg/ml)** |
| --- | --- | --- | --- | --- |
| -7.54 | 1.76 | 1.38 | 1.11 | 0.236 |
| 0.90 | 3.25 | 4.01 | 2.01 | 0.939 |
| 15.15 | 2.05 | 1.40 | 1.02 | 0.227 |
| 16.58 | 1.27 | 3.05 | 1.69 | 0.544 |
| 73.38 | 1.16 | 1.31 | 1.27 | 0.188 |

SUVR_Me_: SUVR in the mesial temporal region of interest. SUVR_Te_: SUVR in the temporoparietal region of interest. SUVR_R_: SUVR in the rest of neocortex.

**Supplementary Table 6. ROC parameters for biological PET stages, *in the whole cohort***

|  | **AUC** | **Youden** | **Sensitivity** | **Specificity** | **PPV** | **NPV** | **Accuracy** |
| --- | --- | --- | --- | --- | --- | --- | --- |
| **A-T-**  ***vs.***  **A+** | 0.94  [0.92 - 0.96] | 0.18 [0.14 - 0.18] | 0.87  [0.85 - 0.95] | 0.90  [0.82 - 0.94] | 0.94  [0.90 - 0.96] | 0.79  [0.75 - 0.90] | 0.88 [0.86 - 0.92] |
| **A-T-, A+T-, A+T_MTL_+**  ***vs.***  **A+T_MOD_+, A+T_HIGH_+** | 0.97 [0.95 - 0.98] | 0.32 [0.32 - 0.41] | 0.95  [0.88 - 0.97] | 0.87  [0.85 - 0.95] | 0.81  [0.77 - 0.91] | 0.97  [0.93 - 0.98] | 0.90 [0.88 - 0.94] |
| **A-T-, A+T-, A+T_MTL_+,**  **A+T_MOD_+**  ***vs.***  **A+T_HIGH_+** | 0.94 [0.91 - 0.96] | 0.44 [0.44 - 0.65] | 0.98  [0.87 - 1.00] | 0.79  [0.77 - 0.91] | 0.44  [0.40 - 0.65] | 1.00  [0.97 - 1.0] | 0.82 [0.80 - 0.91] |

Values are presented as mean [bootstrapped 95% confidence interval]. *A-T-* Aβ negative & tau negative; *A+T-* Aβ positive & tau negative; *A+T_MTL_+* Aβ positive & tau uptake limited to medial temporal region; *A+T_MOD_+* Aβ positive & moderate tau uptake in temporo-parietal region; *A+T_HIGH_+* Aβ positive & high tau uptake in temporo-parietal region

**Supplementary Table 7. ROC parameters for biological PET stages, *in the CI group***

|  | **AUC** | **Youden** | **Sensitivity** | **Specificity** | **PPV** | **NPV** | **Accuracy** |
| --- | --- | --- | --- | --- | --- | --- | --- |
| **A-T-**  ***vs.***  **A+** | 0.97 [0.95 - 0.98] | 0.18 [0.18 - 0.24] | 0.93 [0.84 - 0.96] | 0.93 [0.89 - 1.0] | 0.98 [0.98 - 1.0] | 0.75 [0.57 - 0.85] | 0.93 [0.87 - 0.96] |
| **A-T-, A+T-, A+T_MTL_+**  ***vs.***  **A+T_MOD_+, A+T_HIGH_+** | 0.96 [0.94 - 0.98] | 0.37 [0.33 - 0.46] | 0.91 [0.84 - 0.97] | 0.89 [0.83 - 0.96] | 0.91 [0.87 - 0.97] | 0.89 [0.82 - 0.96] | 0.90 [0.88 - 0.94] |
| **A-T-, A+T-, A+T_MTL_+,**  **A+T_MOD_+**  ***vs.***  **A+T_HIGH_+** | 0.89 [0.85 - 0.93] | 0.63 [0.44 - 0.66] | 0.88 [0.81 - 1.00] | 0.78 [0.64 - 0.86] | 0.57 [0.44 - 0.69] | 0.95 [0.92 - 1.0] | 0.81 [0.73 - 0.87] |

Values are presented as mean [bootstrapped 95% confidence interval]. *A-T-* Aβ negative & tau negative; *A+T-* Aβ positive & tau negative; *A+T_MTL_+* Aβ positive & tau uptake limited to medial temporal region; *A+T_MOD_+* Aβ positive & moderate tau uptake in temporo-parietal region; *A+T_HIGH_+* Aβ positive & high tau uptake in temporo-parietal region.

**Supplementary Figure 1. Plasma pTau217 versus tau PET SUVR (95^th^ percentile-based tau PET thresholds)**


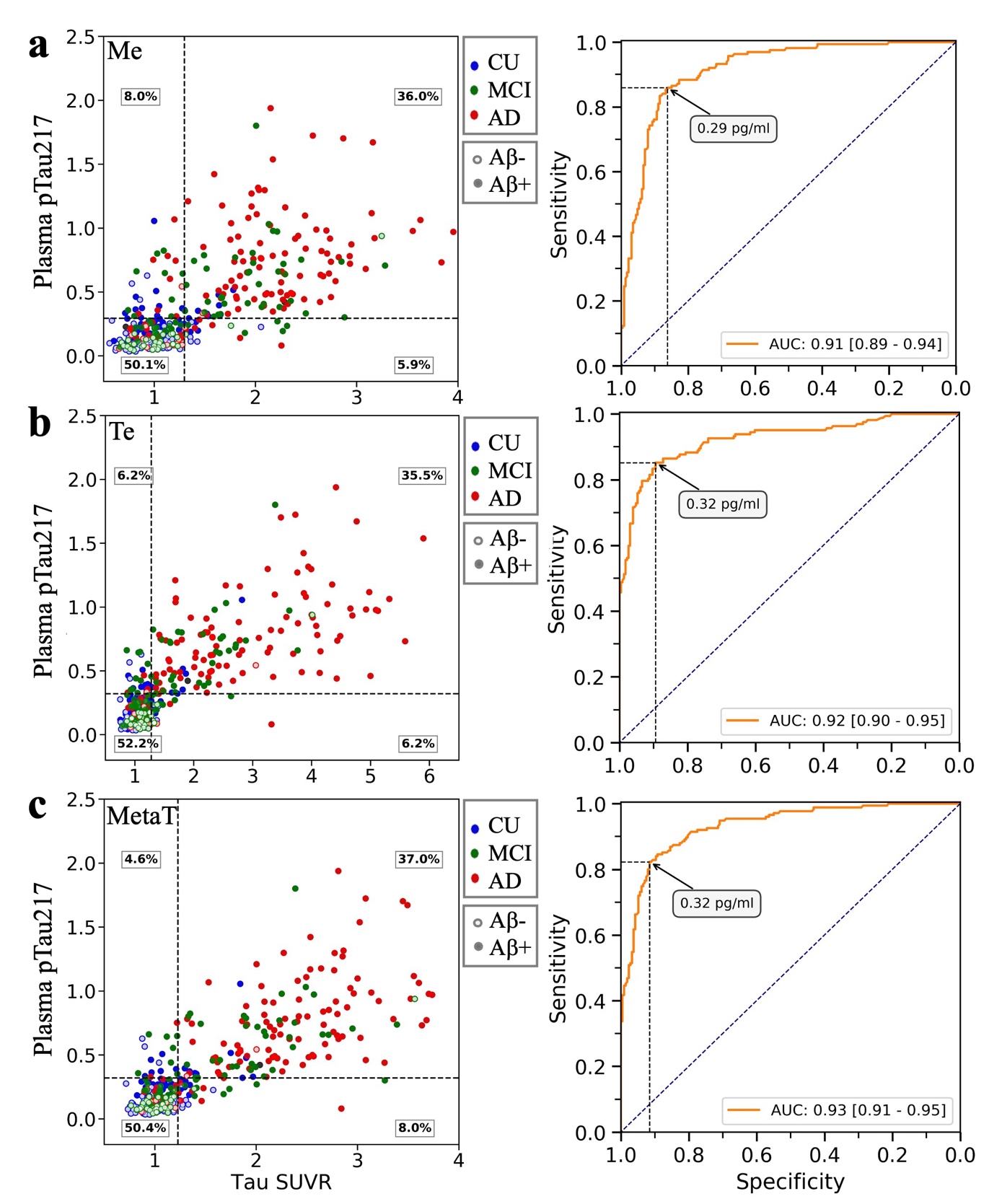


ROC curves and scatter plots of plasma pTau217 versus tau PET SUVR in **(a)** Me, **(b)** Te, and **(c)** MetaT ROI, in the whole cohort (n = 388). AUCs are reported with bootstrapped 95% confidence intervals shown in square brackets. Clinical groups are color-coded: red for dementia (n = 132), green for MCI (n = 100), and blue for CU (n = 156). Solid circles are Aβ PET positive. In the scatter plots, the horizontal dashed line corresponds to the pTau217 threshold derived from the Youden’s index. The vertical dashed line is the tau SUVR threshold, derived from 95^th^ percentile of SUVR values in Aβ negative CU. In the ROC curves, the vertical and horizontal dashed lines represent the sensitivity and specificity values corresponding to the annotated Youden threshold. CU: cognitively unimpaired; MCI: mild cognitive impairment; AD: Alzheimer’s Disease; Me: mesial temporal ROI; Te: temporoparietal ROI, and MetaT: meta temporal ROI.

**Supplementary Figure 2. Discrimination between participants with low tau (Braak 0 – III) and those with high tau (Braak IV-VI)**


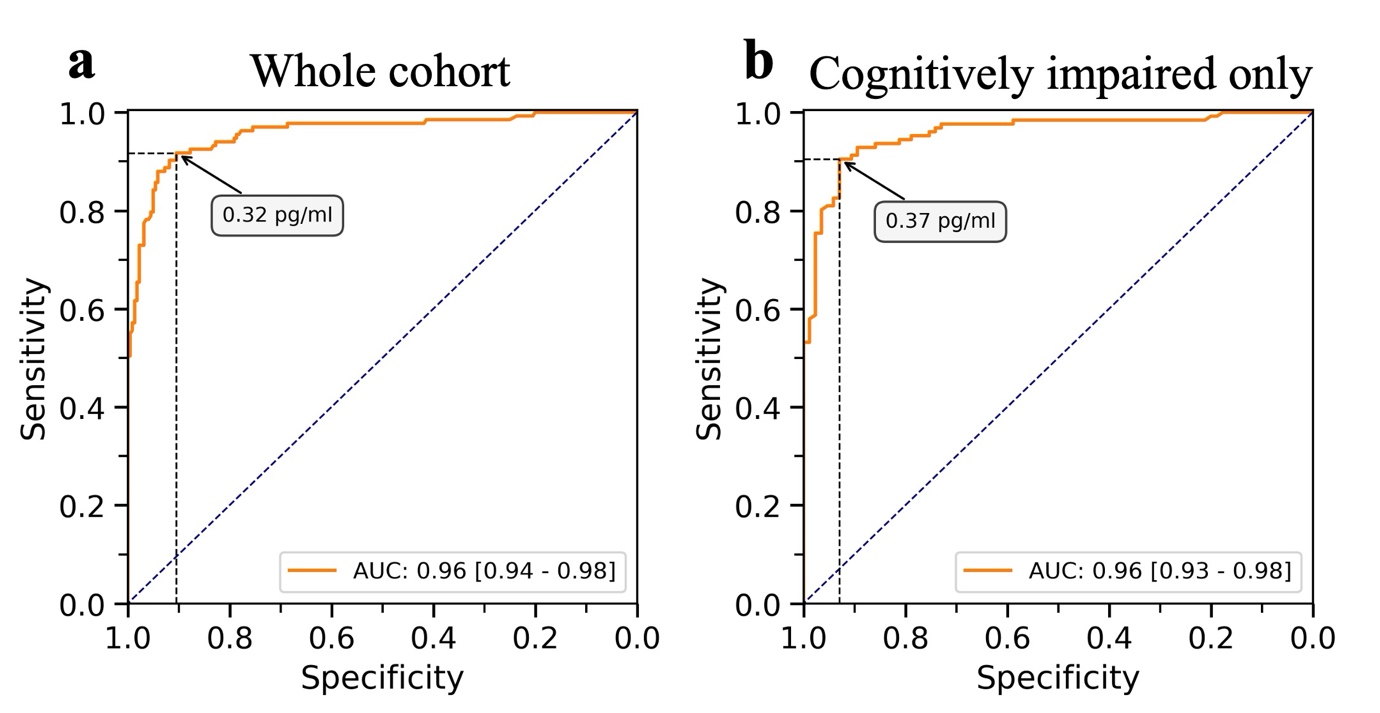


Plasma pTau217 can discriminate between Braak 0-III and Braak IV-VI in the **(a)** whole cohort (n = 388) and **(b)** cognitively impaired (n = 232), with high AUC. Across the whole cohort, the AUC was 0.96 [0.94 - 0.98] and the Youden’s index threshold of 0.32 [0.32 - 0.38] pg/ml gave a sensitivity of 0.92 [0.86 - 0.95], specificity of 0.90 [0.89 - 0.96], PPV of 0.85 [0.82 - 0.93], NPV of 0.95 [0.91 - 0.97] and accuracy of 0.91 [0.89 - 0.94]. In the cognitively impaired, the AUC was 0.96 [0.93 - 0.98] and the Youden’s index threshold of 0.37 [0.33 - 0.38] pg/ml yielded a sensitivity of 0.90 [0.87 - 0.96], specificity of 0.93 [0.87 - 0.98], PPV of 0.95 [0.91 - 0.98], NPV of 0.87 [0.83 - 0.94] and accuracy of 0.91 [0.88 - 0.95].
